# Targeting cell surface glycans with lectin-coated fluorescent nanodiamonds†

**DOI:** 10.1039/d2na00036a

**Published:** 2022-02-07

**Authors:** Mina Ghanimi Fard, Zahra Khabir, Philipp Reineck, Nicole M. Cordina, Hiroshi Abe, Takeshi Ohshima, Sagar Dalal, Brant C. Gibson, Nicolle H. Packer, Lindsay M. Parker

**Affiliations:** School of Natural Sciences, Centre of Excellence for Nanoscale BioPhotonics, Macquarie University Sydney NSW 2109 Australia lindsay.parker@mq.edu.au +61 2 9850 8269; ARC Centre of Excellence for Nanoscale BioPhotonics, School of Science, RMIT University Melbourne VIC 3001 Australia; Quantum Beam Science Research Directorate, The Institute for Quantum Life Science, National Institutes for Quantum Science and Technology Takasaki Gunma 3701292 Japan; Institute for Glycomics, Griffith University Southport QLD 4222 Australia

## Abstract

Glycosylation is arguably the most important functional post-translational modification in brain cells and abnormal cell surface glycan expression has been associated with neurological diseases and brain cancers. In this study we developed a novel method for uptake of fluorescent nanodiamonds (FND), carbon-based nanoparticles with low toxicity and easily modifiable surfaces, into brain cell subtypes by targeting their glycan receptors with carbohydrate-binding lectins. Lectins facilitated uptake of 120 nm FND with nitrogen-vacancy centers in three types of brain cells – U87-MG astrocytes, PC12 neurons and BV-2 microglia cells. The nanodiamond/lectin complexes used in this study target glycans that have been described to be altered in brain diseases including sialic acid glycans *via* wheat (*Triticum aestivum*) germ agglutinin (WGA), high mannose glycans *via* tomato (*Lycopersicon esculentum*) lectin (TL) and core fucosylated glycans *via Aleuria aurantia* lectin (AAL). The lectin conjugated nanodiamonds were taken up differently by the various brain cell types with fucose binding AAL/FNDs taken up preferentially by glioblastoma phenotype astrocyte cells (U87-MG), sialic acid binding WGA/FNDs by neuronal phenotype cells (PC12) and high mannose binding TL/FNDs by microglial cells (BV-2). With increasing recognition of glycans having a role in many diseases, the lectin bioconjugated nanodiamonds developed here are well suited for further investigation into theranostic applications.

## Abstract 2

Nanodiamonds were coated in lectins to target glycan receptors on astrocytes, neurons and microglia. The uptake in each cell type was variable depending on their coating of *Aleuria aurantia* lectin, wheat germ agglutinin or tomato lectin.

## Graphical abstract

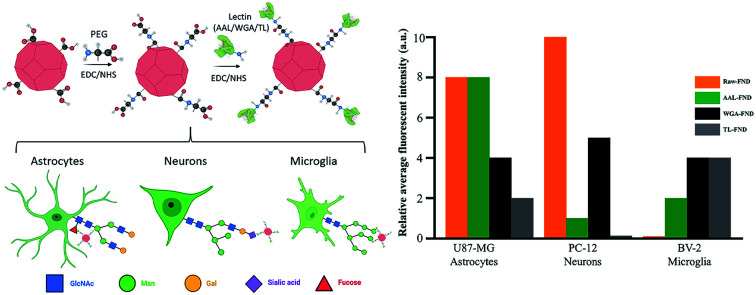

## 1 Introduction

Fluorescent nanodiamonds (FNDs) are highly biocompatible *in vitro* and *in vivo* carbon based nanocarrier tracking agents suitable for drug delivery^1,2^ including for anti-cancer therapeutics.^3–6^ They have excellent photostability and show high optical contrast in fluorescence microscopy images^1,7–9^ and the highest cellular uptake among members of the nanocarbon family,^9,10^ making them suitable for bioimaging and diagnostics. The surface chemistry of FNDs allows for surface functionalization with various functional groups (*e.g.* carboxylic groups) that facilitate attachment of different molecules,^11^ and FND particle sizes can be tailored easily within the size range of 20–500 nm.^12,13^ The fluorescent diamonds contain defects in their crystal lattice that are key contributors to their luminescence and photostability by trapping photoelectrons and energy in the center of the lattice surrounded by carbons following photoemission.^14,15^ Nitrogen-vacancy (NV) center nanodiamonds are most commonly used for research purposes as they show broad fluorescence in the spectral range between 600 nm and 800 nm in the first near-infrared biological window.^16^

Selective targeting of only diseased cells and exclusion of targeting to healthy cells is an important and emerging field of study in applications of nanodiamonds for drug delivery.^17–20^ Strategies for attaching a variety of polymer coatings and antibodies to FNDs have been developed in recent years, although the majority of research in this field has been done on detonation FNDs rather than high pressure–high temperature (HPHT) FNDs, which were used in this study.^6,11,21^ Functionalized nanodiamonds have been targeted to a variety of diagnostic cancer biomarkers on cellular membranes, such as CD44, for improving bioimaging and compatibility with correlative light and electron microscopy.^21,22^ Additionally, intracellular nanodiamond targeting strategies developed for organelles *via* monoclonal anti-nuclear pore complex antibody,^23^ mitochondrial localizing sequence (MLS) peptide^24^ and nuclear localization signal (NLS) peptide^25^ will be advantageous for improving long term imaging, localized magnetometry, monitoring of intracellular temperature changes and delivery of therapeutic antisense agents.

An unexplored strategy for targeting cells with nanodiamonds is to attach lectins, a class of proteins with affinity for binding to glycans.^26,27^ Glycans, located at the outermost surface of cells, are a first point of contact for lectins^28^ and almost all cells are coated with a ∼150 nm thick layer of glycoconjugates known as the glycocalyx.^29,30^ Glycosylation is a post translational modification that attaches glycan moieties to proteins, proteoglycans and lipids^30^ and changed glycosylation is evident in many brain diseases and disorders^31^ making the targeting of cell surface glycans an attractive prospect.^30^ Binding of lectins to glycans has been shown to initiate cellular uptake *via* receptor-mediated endocytosis, causing internalization of the lectins.^19,32^ Glycan coated detonation nanodiamonds targeting bacterial lectins have been developed previously to help inhibit biofilm formation and bacterial colonization^33,34^ but to date, no studies have utilized lectin coated nanodiamonds for glycan biomarker directed targeting or uptake. There are lectins with specific binding sites for a single type of glycan receptor such as *Aleuria aurantia* lectin (AAL), which binds specifically to core fucose (the monosaccharide 6-deoxy-l-galactose) on protein-linked *N*-glycans,^35,36^ while other lectins have binding pockets for more than one glycan structural type.^37^ Wheat germ agglutinin (WGA), for example, can multivalently bind to sialic acid and/or *N*-acetylglucosamine (GlcNAc) containing glycans.^38^ Similarly, tomato lectin (*Lycopersicon esculentum*; TL) has wide specificity for GlcNAc,^39^ as well as poly-*N*-acetyl lactosamine (GlcNAcβ1-4Gal)_*n*_ (ref. 40) and oligomannose *N*-glycan structures.^41^

Glycan expression is well known to be altered in the central nervous system (CNS) cells throughout developmental stages and CNS disease progression^30,42,43^ but targeting specific cells *via* their glycan receptors with lectin-conjugated nanodiamonds based on cell-type dependent expression is so far unexplored. For example, sialic acid is ubiquitously expressed on CNS cells and changes in expression levels have been observed in the pathophysiology of Alzheimer disease, Parkinson's disease, schizophrenia,^44^ inclusion body myopathy^45^ and glioblastoma.^46^ Additionally, core fucosylated *N*-glycans are a viable biomarker for CNS specific cancers such as glioblastoma multiforme, which express increased core fucose structures on the surface of cancer cells and tissues compared to normal brain cells and tissues and therefore core fucosylated *N*-glycans could be a target for diagnosis and drug delivery.^47,48^

The glycan receptor subtypes occurring on CNS cell surfaces can potentially be cell-type specific and altered during CNS development and disease.^49–51^ The current study is reporting on the first strategy for conjugating nanodiamonds to different lectins and imaging the targeting of specific CNS cell types. This bioconjugation was carried out using carbodiimide chemistry and was characterized by dynamic light scattering, fluorescence microscopy and transmission electron microscopy (TEM). The uptake of the three types of lectin-conjugated nanodiamonds was evaluated in neuronal cells, microglia cells and astrocyte cells by laser scanning confocal fluorescence microscopy and we report on cellular health, active uptake and cell viability of the exposed cell types. Our findings demonstrate that lectin-conjugated nanodiamonds are compatible with bio-imaging studies in brain cells and are endocytosed under normal culturing conditions, these coated diamonds show CNS cell-type preferences for uptake based on their lectin binding affinities. Delivery of most nanomaterials or drugs into the CNS is often blocked by the blood–brain barrier (BBB) as a protection mechanism^52^ although polyethylene glycol (PEG) and biopolymer coating on nanodiamonds facilitates their passing through the BBB following intravenous injection in mice.^53^ Coating FNDs with PEG and lectins,^54^ especially WGA, could be even more beneficial for crossing the BBB^55^ without causing disruption,^56^ while achieving targeting to CNS cells. The nanodiamond-based platform developed here can be adapted in future studies for efficient ways of achieving cell-type specific drug delivery to brain cells using lectin-conjugated nanodiamonds.

## 2 Experimental

### 2.1 Production of carboxylated nanodiamonds

Nanodiamonds produced *via* the HPHT synthesis with a broad size distribution of 30–150 nm were purchased from Nabond Technologies, China. Nitrogen-vacancy centers were created through electron beam irradiation (2 MeV, fluence of 1 × 10^18^ electrons per cm^2^) at room temperature followed by annealing in vacuum for 2 hours at 800 °C and oxidized for 4.5 hours in air at 520 °C. The oxidized nanodiamond powder was dispersed in deionized (DI) water at 1 mg mL^−1^ and sonicated using a horn sonicator with a 66% duty cycle for 1 hour at 125 W. As a result of the of the oxidation process several oxygen-containing groups are present on the surface of FNDs, which was shown in the Fourier-transform infrared (FTIR) results as explained in Section 2.2.

### 2.2 Bioconjugation of fluorescent nanodiamonds with lectins

Wheat germ agglutinin (WGA, L4895), tomato lectin (TL, L0401), fluorescein isothiocyanate conjugated WGA (WGA–FITC) (L4895), fluorescein isothiocyanate conjugated TL (TL–FITC) (L0401), 1-ethyl-3-(3-dimethylaminopropyl)carbodiimide (EDC, 03450), *N*-hydroxysuccinimide (NHS, 130672), ethanolamine ≥99% (E6133), dithiothreitol (DTT), phosphate buffered saline with 10% (v/v) bovine serum albumin (BSA containing PBS, SRE0036), Dulbecco's phosphate buffered saline (PBS, Merck; D8537; 1×) and poly(ethylene glycol) 2-aminoethyl ether acetic acid (PEG, 757861), a modified version of PEG containing carboxyl and amine groups, were purchased from Sigma-Aldrich, Australia. *Aleuria aurantia* lectin (AAL, L-1390L) and fluorescein isothiocyanate conjugated AAL (AAL–FITC) (FL-1391) were purchased from Vector Laboratories, United States.

**Fig. 1:**
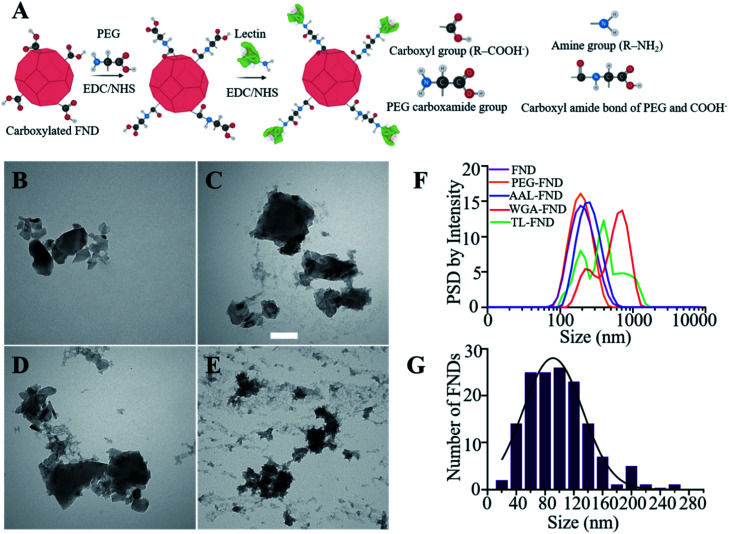
(A) Schematic representation of EDC/NHS chemistry to bioconjugate carboxylated FNDs to lectins using PEG (poly(ethylene glycol) 2-aminoethyl ether acetic acid). (B), (C), (D) and (E) are stained (UAR negative staining) TEM images of raw, AAL–, WGA– and TL–FNDs, respectively (no BSA added). Scale bars 100 nm. (F) Particle size distribution (PSD) by intensity of colloidal dispersion of raw and lectin bioconjugated FNDs in water after sonication. (G) Particle size distribution histogram of raw FNDs as supplied with an average size of 100 nm.

Each lectin was conjugated separately to the surface of FNDs following a procedure we have previously described that was applied to conjugating antibody or streptavidin to FNDs.^8^ Briefly, 400 μL of 120 nm FNDs (1 mg mL^−1^, FND powder weighed and suspended in DI water) were first PEGylated with 24 μL of 50 mg mL^−1^ PEG_22_ using EDC/NHS esterification. 80 μL of 20 mg mL^−1^ EDC in acidic water (pH 4) that was made from adding 5 mL hydrochloric acid to 45 mL DI water and 120 μL of 20 mg mL^−1^ NHS in acidic water (pH 4) were incubated at room temperature on a shaker for 20 minutes and EDC was inactivated by addition of 20 μL of 1 M DTT. The carboxylic groups on the surface of FNDs reacted with amine groups on the PEG (poly(ethylene glycol) 2-aminoethyl ether acetic acid) to form an amide bond between the nanodiamond and the PEG. After capping FNDs with PEG, the same amounts of EDC/NHS in acidic water were added again to activate the carboxyl groups on the PEG (poly(ethylene glycol) 2-aminoethyl ether acetic acid) and the same incubation on the shaker with same conditions was repeated, which then enabled binding to the amine groups on the lectins (Fig. 1A). The bioconjugation included two separate steps each including 3–4 hours of incubation on a shaker at room temperature after addition of PEG/lectins, and an overnight incubation on shaker at 4 °C, followed by quenching with 20 μL of 1 M ethanolamine to react with any remaining NHS esters on FNDs, on the next day and incubation on the shaker for two hours at room temperature. After that, each solution was centrifuged (20 000×*g*, 15 minutes) to remove excess PEG/lectin and ethanolamine. Finally FND–PEG/FND–PEG–lectin pellet was resuspended in neutral DI water (pH 7). These final solutions were bath-sonicated at 300 W for 10 minutes after bioconjugation to reduce unwanted aggregation. An additional bioconjugation batch was made in which FITC-labelled lectins of each type were also conjugated to FNDs in the same way to assess their successful binding and stability.

BSA in PBS was added to each bioconjugated FNDs to block non-specific binding and prevent aggregation by increasing colloidal stability.^57^ BSA was added until each solution reached a concentration of 1 mg mL^−1^ as a standard, which was the initial concentration of raw FNDs for comparison purposes (Fig. S1,† the standard curve created to read concentrations with a NanoDrop instrument). To confirm that the solution had a 1 mg mL^−1^ concentration, dry-weighing of 10 μL of the lectin bioconjugated FNDs with a precision balance (Secura613-1S, Sartorius, Germany) was done and replicated 3 times, and a dried weight of ∼0.01 mg confirmed the correct concentration of 1 mg mL^−1^ of bioconjugated FNDs. Another batch of nanodiamonds were bioconjugated with lectins using the same methods explained above, but without BSA addition for experiments that measured the amount of proteins on FNDs, in order to exclude measurement of BSA proteins and only include lectins in the calculations. They were diluted by PBS that did not contain any BSA to reach the 1 mg mL^−1^ standard concentration.

### 2.3 Characterization of lectin-bioconjugated nanodiamonds

A brightfield TEM (CM10, Philips, Netherlands) operated at the accelerating voltage of 100 keV was utilized to determine the size of FNDs and visualize the lectin coating. For TEM sample preparation, raw and bioconjugated FNDs in aqueous solutions, from the batch that did not include bovine serum albumin (BSA), were deposited on 300 mesh formvar/carbon film copper grids (FCF300-Cu, Electron Microscopy Sciences, USA) followed by uranyl acetate replacement or UAR (22405, Electron Microscopy Sciences, USA) negative staining. For staining, UAR was diluted with DI water (1 : 100) and drops of the diluted solution were placed on parafilm. Then, grids were placed on each drop and incubated at room temperature. After 40 minutes, excess stain was removed by blotting the grids with filter paper, then were washed in DI water and air-dried overnight.

Particle size distribution, polydispersity index was measured using DLS (Zetasizer NS, Malvern, U.K.) with a 633 nm laser source. For sample preparation, 4 μL of raw and bioconjugated FND solutions were diluted in 1 mL of DI water and bath sonicated at 300 W for 10 minutes and placed in a folded capillary zeta cell (DTS1070, Malvern, UK). To compare the effect of bath sonication on the aggregation of FNDs, the measurements were repeated also without 10 minutes of bath sonication at 300 W for 10 minutes before sample preparation for uptake experiments (Fig. S3 and S4†).

A Fourier-transform infrared spectrum of the FNDs before bioconjugation (Fig. S5†) was recorded with a Frontier spectrometer (PerkinElmer, USA) fitted with attenuated total reflection (ART) attachment. Fluorescence spectra of particles suspended in DI water (0.1 mg mL^−1^) were recorded with a custom-built fluorescence spectroscopy setup using 520 nm laser excitation of the FNDs and a spectrometer (SpectraPro, Princeton Instruments, USA) fitted with a charge-coupled device camera (PIXIS, Princeton Instruments, USA) described in detail elsewhere.^58^

To confirm the bioconjugation of lectins to FNDs, FITC labelled lectins were bioconjugated to FNDs without BSA (Section 2.2) and 10 μL of raw FND and each of the FITC–lectin–FNDs were drop casted with a pipette onto a glass slide and covered by a square coverslip, incubated in the dark overnight at room temperature to dry and imaged by the FV3000 confocal microscope.

Quantification of lectins conjugated to FNDs was carried out using the micro bicinchoninic acid based (Micro BCA™) Protein Assay Kit (23235 Thermo Fisher Scientific). Briefly, following the protocol provided by supplier for a microplate assay, bovine serum albumin (BSA) standard samples with varying concentrations of 5–200 μg mL^−1^ were prepared for the calibration. The samples prepared separately were lectin bioconjugated FNDs without BSA or raw FND solutions at the same concentration (0.6 mg mL^−1^). 150 μL of each control sample or test sample were transferred to a flat bottom 96-well plate (Greiner CELLSTAR®), and 150 μL of Micro BCA reagent added to each well. The microplate was incubated at 37 °C. After 2 hours, the absorbance was measured at 562 nm on a PHERAstar® FSX plate reader (BMG LABTECH, Germany). Finally, the concentration of lectin protein present in each solution was calculated using the standard curve and validated by the dry weight method explained in Section 2.2.

### 2.4 Cell culture

Three cell lines were utilized representing different types of brain cells: rat PC12 neuronal phenotype cells, human U87-MG glioblastoma phenotype astrocyte cells and mouse BV-2 microglia phenotype cells. PC12 cells were obtained from ATCC (CRL-1721), U87-MG from ATCC (HTB-14), and BV-2 cells were a generous gift from Professor Gilles Guillemin (Macquarie University, Sydney, Australia). These cell lines were each grown in complete Dulbecco Modified Eagle Medium (DMEM) with high glucose (4500 mg L^−1^), 10% (v/v) Fetal Bovine Serum (FBS) (10437028 Gibco Life Technologies, USDA-approved regions), and 1% (v/v) penicillin–streptomycin (Pen/strep) (10 000 units penicillin and 10 mg streptomycin per mL in 0.9% (v/v) NaCl, P0781 Merck) with the addition of 5% (v/v) Normal Horse Serum (H1138 Merck; Australian Origin) only for PC12 cells as recommended by American Type Culture Collection (ATCC). Cells were maintained in a humidified incubator with a mix of 95% air and 5% CO2 gas at 37 °C. Cells were cultured aseptically in Greiner Bio-One Cellstar© T75 tissue culture flasks, sterile with plug seal caps (Mfg part number: 658170) in biosafety cabinets. All cells were sub-cultured for further experiments.

### 2.5 Treatment of cells with bioconjugated nanodiamonds and actin staining

A stock solution was prepared containing either 10 μg of raw FNDs (non-bioconjugated) or lectin-conjugated nanodiamonds per 1 mL of PBS. Cells at a seeding density of 50 000/well were sub-cultured onto sterile coverslips (no. 1, 22 × 22 mm^2^ MenzelGläser) in 6 well plates for 24 hours. Afterwards, cells, except for untreated controls (Fig. S2†) were treated with 10 μL of a 1 mg mL^−1^ stock solution of either raw or bioconjugated nanodiamonds at 37 °C and 5% CO_2_ for 24 hours. Media was removed, cells were washed with 1× PBS prior to 10 min fixation on a shaker with 4% (v/v) formaldehyde solution (F8775, Merck, diluted in Milli-Q water) and then washed a further 3 times with 1× PBS. The fixed cells on coverslips were stained with f-actin filament stain according to the manufacturer's protocol (ActinGreen™ 488 ReadyProbes™ reagent R37110, Life Technologies, Australia) in 1 mL of PBS, incubated on a rocker for 30 min, and washed again 3 times with PBS. Coverslips were then mounted onto microscopy slides (Livingstone, Universal Microscope Glass Slide) using ProLong™ Glass antifade mountant with NucBlue™ (P36981, Life Technologies, Australia). These protocols were repeated to produce *n* = 3 replicate slides for each condition for analysis.

### 2.6 Lipopolysaccharide-stimulated inflammation in BV-2 microglia cells

Resting microglia (BV-2) cells generally do not have high uptake of nanoparticles^59^ unless they are activated by a signal such as lipopolysaccharide (LPS)-stimulated inflammation.^60^ As a control for uptake, 5000 BV-2 microglia cells were sub-cultured in complete DMEM overnight onto sterile coverslips (22 × 22 mm^2^) in 6-well plates as described in Section 2.5. Cells were treated with LPS (500 ng mL^−1^; LPS-EB Ultrapure; InvivoGen) and incubated for 4 hours; cells were then washed with 1% (v/v) PBS and 10 μg of raw–FNDs (1 mg mL^−1^) in 2 mL of complete media and incubated at 37 °C and 5% CO_2_ for 24 hours. Then, cells were washed with 1% (v/v) PBS, fixed and mounted on microscopy slides following the protocol described above.

### 2.7 Cytotoxicity assays

CellTiter 96® AQueous One Solution Cell Proliferation Assay (MTS) kit (3-(4,5-dimethylthiazol-2-yl)-5-(3-carboxymethoxyphenyl)-2-(4-sulfophenyl)-2*H*-tetrazolium, inner salt; MTS) was used to measure the viability of cells after treatment by different concentrations of raw/bioconjugated FNDs. They were then seeded at densities of 10000 (U87-MG), 5000 (BV-2) or 25000 (PC12) cells/well into 96 well plates (Greiner CELLSTAR®) with 200 μL complete DMEM media and kept in a humidified incubator with 95% air and 5% CO_2_ at 37 °C overnight. Each of the above cell lines reached confluency in the same size dish within a different period of time. Therefore, cell seeding densities of each cell type were optimized for confluency in 24 hours prior to MTS assay in order to avoid the saturation of absorbance. Cells were treated with raw/bioconjugated FNDs suspensions at concentrations of 5, 10, 15 and 30 μg mL^−1^ with 5 replicates. Cells without any FNDs (0 μg mL^−1^) in complete cell culture media were used as negative controls (shown as untreated control). Raw/bioconjugated FND solutions (3 replicates) were added to complete cell culture media at concentrations of 0, 5, 10, 15 and 30 μg mL^−1^ but without any cells and were defined as blanks. After 24 hours of incubation with 95% air/5% CO_2_ at 37 °C, 20 μL MTS reagent was added to each well. After 1 hour, absorbance was measured at 490 nm wavelength using a microplate reader (Synergy H1, Biotek, USA). GraphPad Prism 9 software was used to analyze the cell viability by normalizing the mean absorbance of treated cells compared to the control of each raw or bioconjugated FNDs concentration after subtraction of blank samples.

### 2.8 Low temperature uptake

Cell viability of U87-MG cells at 4 °C was tested for 8 hours. Approximately 1000 U87-MG cells were sub-cultured in 3 wells of a 24 well plate (×3 replicates) with complete media (DMEM, 10% (v/v) fetal bovine serum and 1% (v/v) Pen/strep) for 24 hours. The medium was then changed to HEPES containing phenol red-free DMEM (21063029, Gibco, Life Technologies), known for maintaining physiological pH when 95% air/5% CO_2_ condition is not available,^61^ 10% (v/v) FBS and 1% (v/v) pen/strep. After changing the medium, the cells were incubated at 4 °C for 8 hours. Muse™ Cell Analyzer and Viability Assay Kit (MCH100102) benchtop flow cytometry was used to calculate the percentage of viable U87-MG cells in each sample.

U87-MG astrocyte cells were sub-cultured onto round coverslips (Livingstone, CS12RD) within 2 × 24 well plates (Greiner CELLSTAR®, 662160) for 24 hours. Complete medium was added to the cells of one plate and the second plate of cells were treated with HEPES containing DMEM. The cells, except for untreated controls (cells with no FNDs), were then treated with 10 μg of raw–FND or bioconjugated FND 1 mg mL^−1^ solutions per well ×3 replicates in each plate. The plate with complete media (control) was incubated at 37 °C and 95% air/5% CO_2_ for 8 hours and the plate with HEPES containing DMEM (experiment) was incubated at 4 °C for 8 hours. The medium was removed, cells were fixed, and stained with ActinGreen and mounted onto slides following the protocol described in Section 2.5. These samples were imaged by fluorescent widefield inverted microscopy (Olympus IX83, with DP80 monochrome camera).

### 2.9 Light microscopy

An Olympus Fluoview (FV3000) confocal laser scanning microscope equipped with a UAPON 100x/1.49 NA TIRF oil objective lens with Olympus Low Autofluorescence Immersion Oil (IMMOIL-F30CC, part #86-834) was used to confirm that FNDs were successfully bioconjugated to FITC–lectins (by co-localization, see details on image analysis below) *via* laser excitation and emissions collected for FITC (ex 488 nm; em 500–550 nm) and nanodiamond (ex 561 nm; em 650–750 nm). Cells containing nanodiamonds were imaged on the same microscope using a 60× long working distance objective (UPLSAPO60XS2; W.D.: 0.3 mm, silicon oil) with Olympus silicone immersion oil (part # Z-81114), and a scanning speed of 8.0 μs per pixel capturing 10 Z-slices with 0.5 μm step size from each image. The resulting images were used for analysis by measuring FND fluorescence intensity. FNDs, NucBlue™ and ActinGreen™ stains were excited at 561 nm, 405 nm and 488 nm, respectively, and their fluorescence was collected at 650–750 nm, 420–450 nm and 500–550 nm, respectively.

An Olympus IX83 widefield fluorescence microscope (Olympus, with DP80 monochrome camera) equipped with a PLAPON O 60x/1.42 NA, oil objective lens with Olympus Low Autofluorescence Immersion Oil (IMMOIL-F30CC, part #86-834) was utilized for rapid imaging of the low temperature uptake experimental samples in order to determine passive uptake of nanodiamond/lectin complexes using staining of NucBlue (ex 358 nm; em 455 nm), ActinGreen™ (ex 494 nm; em 518 nm) and a custom installed filter suitable for NV center fluorescent nanodiamonds: 555 nm excitation 25 nm bandwidth (ET555/25x Chroma); 635 nm dichroic (T635lpxr Chroma); 700 nm emission 75 nm bandwidth (ET700/75m Chroma).

### 2.10 Image analysis

For measuring co-localization of FITC-labelled lectins with FNDs, a multi-channel object-based approach to calculate the co-localized regions was used by Imaris image analysis (version: 8.0.2) software.^62^ Two color channels were analyzed for overlapping regions in order to identify the intensity of the co-localized regions. The object channel (FND) was overlapped on the background channel (FITC) each with a different arbitrary colour, manually creating a third channel containing the filtered voxels from the co-localized sites. The intensity from these filtered regions was used to calculate the percentage of co-localized sections.

For the comparison of the amount of FNDs taken up by each cell line, Imaris image analysis software was utilized measuring the fluorescence intensity of FNDs in the confocal microscopy images of cells. For analysis of cellular uptake, manual thresholding of 10 randomly selected cells from 3 images of each condition (*n* = 30 total cells analyzed per lectin) was performed. A contour surface was created around the borders of each randomly selected cell in the first and last slice of maximum Z projection. The mean fluorescent intensity of all pixels of each cell in the FND channel was collected for data analysis, which was shown with the arbitrary unit (a.u.) by the software. The same process was performed for quantifying the cellular uptake at 37 °C and 4 °C by analyzing the widefield microscopy images.

### 2.11 Statistics

Data analysis and plotting were performed in GraphPad Prism 9 including normality and lognormality tests to verify the normal distribution of data. The data was found to have a non-normal distribution by the Shapiro–Wilk test, therefore statistical significance of data was determined by the non-parametric Kolmogorov–Smirnov test for all experiments. The standard error of mean (SEM) of analyzed samples was represented as error bars. Significance levels are: *p ≤ 0.05, **p ≤ 0.01, ***p ≤ 0.001, and ****p ≤ 0.0001, and not significant for *p* > 0.05.

### 2.12 Figures

Schematic figures in this manuscript including graphical abstract were created by http://Biorender.com website and edited by Microsoft PowerPoint and Windows Paint tool.

## 3 Results

### 3.1 Characterization of lectin–PEG–FND bioconjugates

Before the bioconjugation of FNDs to lectins (Fig. 1A), raw (unconjugated) FNDs were characterized by TEM, showing a normal size distribution ranging from 25 nm to 225 nm size peaking at 100 nm of 100 randomly selected FNDs from six images (Fig. 1B and G). Raw (Fig. 1B) and bioconjugated FNDs showed jagged irregular clumped shapes with layers of protein on the PEG coated FNDs with all lectin conjugations: AAL (Fig. 1C), WGA (Fig. 1D) or TL (Fig. 1E). DLS particle size distribution (PSD, intensity weighted) of raw FNDs, PEG–FNDs and AAL–FNDs each featured only one peak, while conjugated WGA–FNDs and TL–FNDs PSDs displayed multi-peak patterns, which indicate a broad size distribution (Fig. 1F). For raw nanodiamonds and PEG–FNDs the intensity peak at ∼190 nm was observed with a size range between ∼60 nm and ∼500 nm. For all samples, most particles were between 100 and 500 nm in size except for WGA–FNDs which ranged between ∼100 nm and ∼1100 nm. The results of DLS suggest that bioconjugation of raw–FNDs to lectins caused aggregation of particles in the solution which was more noticeable in cases of WGA– and TL–FNDs. The TEM images of raw/bioconjugated FNDs (Fig. S3A–H†) showed that sonication of nanodiamonds for 10 minutes breaks the large aggregated FND particle clusters within an area of ∼400 nm^2^ into smaller aggregates with lengths and widths of less than 100 nm. DLS results before and after bath sonication also confirmed this result (Fig. S4†).

**Fig. 2:**
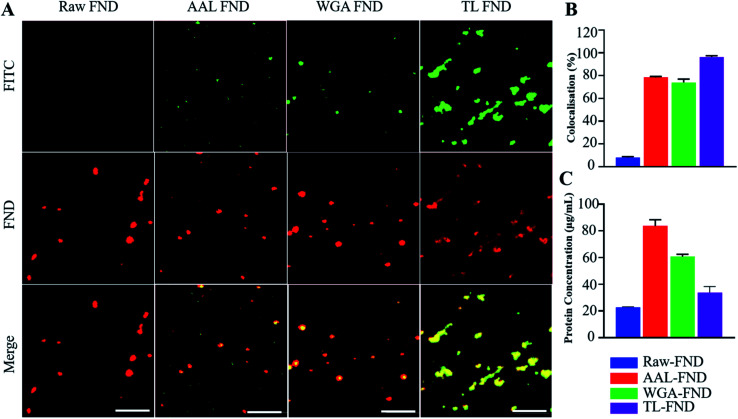
(A) Confocal microscopy images of raw–FNDs (negative control) and lectin–FITC conjugated FNDs at 100× magnification. Colocalization (yellow; bottom row) is shown of FND fluorescence (red; middle row) with FITC fluorescence of conjugated lectins (green; top row) as a demonstration of successful bioconjugation of lectin to FNDs. Scale bars 5 μm. (B) The object channel (FND) was overlapped on the background channel (FITC) each with respectively red and green arbitrary colour manually creating a third channel (merge) containing the filtered voxels from the co-localized sites. The intensity from these filtered regions was used to calculate the percentage of co-localized sections (*n* = 3 images from different areas of samples with magnification of 100× for each condition), which is significantly higher (*p* < 0.0001 in all cases) than raw diamonds for AAL–FND, WGA–FND and TL–FND. (C) The concentration of protein in lectin–FND solutions measured by micro BCA assay is significantly higher for AAL–FND (*p* < 0.0001), WGA–FND (*p* < 0.0001) and TL–FND (*p* < 0.05) than on the surface of raw nanodiamonds obtained by paired *t*-tests.

FNDs conjugated with FITC-labelled lectins exhibited co-localized staining of FITC and nanodiamond emissions as determined by fluorescence microscopy. Fig. 2A shows raw–FNDs as a negative control, with AAL–FITC–FND, WGA–FITC–FND, TL–FITC–FND bioconjugates in yellow (right panels) resulting from the merged channels of green (for FITC; top panels) and red (for FNDs; center panels) where there is colocalization (yellow; bottom panels). Fig. 2B indicates the fluorescence intensity comparison of FNDs (red, em ∼690–800 nm) co-localizing with FITC channel (green em ∼510–540 nm). There was ∼78% of AAL–FITC, ∼73% of WGA–FITC and ∼97% of TL–FITC co-localized with FNDs after the bioconjugation process, which is significantly higher than the ∼8% co-localization seen in the same emission channels with raw–FNDs (negative control, *p* ≤ 0.0001). Micro BCA protein assay revealed the concentration of lectins in AAL–FND, WGA–FND and TL–FND samples were 83.6 μg mL^−1^, 60.6 μg mL^−1^ and 33.7 μg mL^−1^, respectively (Fig. 2C). We observed a slight interference of wavelength absorbance by raw–FNDs, which is shown on the graph for comparison.

### 3.2 Cell health and viability following FND and lectin–FND treatment

**Fig. 3:**
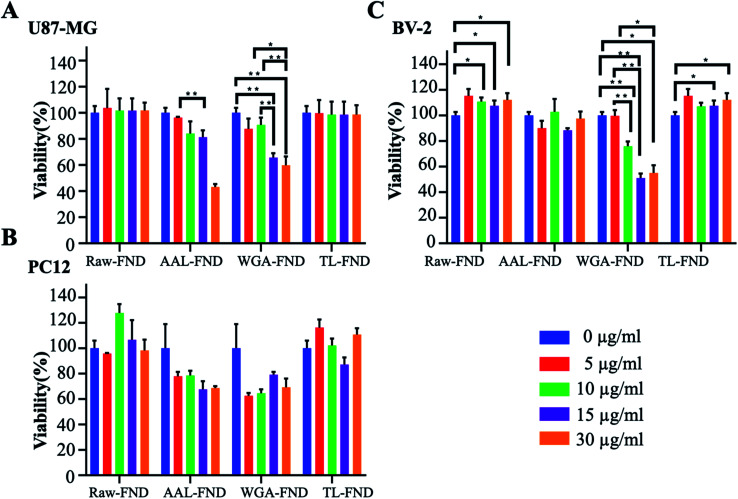
Colorimetric MTS cell proliferation assays (*n* = 5). (A), (B) and (C) cell proliferation viability following treatment with increasing concentrations of each raw or lectin bioconjugated FNDs in glioblastoma astrocytes (U87-MG), neuronal phenotype cells (PC12), and microglial cells (BV-2). Absorbance readings were normalized to the mean values obtained for untreated cells. * for *p* ≤ 0.05, ** for *p* ≤ 0.01, obtained by non-parametric Kolmogorov–Smirnov tests.

Normalized MTS results showed that, in general, treatment with raw/bioconjugated FNDs in concentrations of 10 μg mL^−1^ and below, do not bring cell viability of U87-MG (astrocytes), PC12 (neurons) and BV-2 (microglia) cells lower than 70% compared to untreated control cells (Fig. 3A–C). Interestingly, raw–FND and TL–FND increased cell viability up to 120% compared to untreated cells in all of the three cell lines, especially BV-2 microglia (*p* ≤ 0.05). In contrast, treatment with WGA–FND decreased cell viability in all of the cell lines. Even low concentrations of WGA–FND decreased the viability of PC12 cells to 60% (5 and 10 μg mL^−1^) although the change is not statistically significant (*p* = 0.079 and *p* = 0.28 respectively) while high concentrations (15 μg mL^−1^ and higher) reduced the viability of BV-2 cells significantly by up to ∼50% of untreated control cells (*p* ≤ 0.01). AAL–FNDs at 15 μg mL^−1^ and higher concentrations also decreased cell viability of PC12 (*p* = 0.57) and U87-MG (*p* = 0.079) cells, while they were less harmful to BV-2 cells even at the 30 μg mL^−1^ concentration. Cells that were treated with 10 μg mL^−1^ and lower concentrations of AAL–FNDs showed >80% viability (Fig. 3A–C).

### 3.3 Cellular uptake

**Fig. 4:**
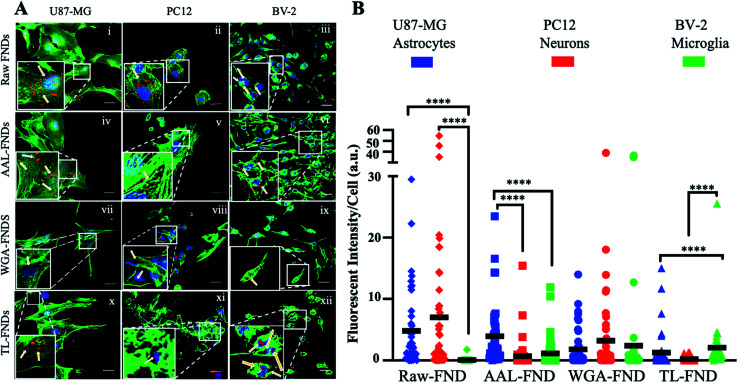
(A). Uptake of raw or lectin bioconjugated FNDs by brain cell subtypes. Confocal microscopy of U87-MG (left column), PC12 (middle column) and BV-2 (right column) cells treated with raw FNDs (first row), AAL–FNDs (second row), WGA–FNDs (third row) and TL–FNDs (fourth row). ActinGreen stain for f-actin filaments (green – ex 488 nm; em 500–550 nm), NucBlue stain of nuclei (blue) and FND fluorescence (red – ex 561 nm; em 650–750 nm) were observed. Inset zoomed areas selected from representative cells highlight FND uptake (indicated by arrows). Scale bars = 20 μm. (B) Quantification of raw and lectin bioconjugated FND uptake by U87-MG (blue), PC12 (red) and BV-2 cells (green) by fluorescence intensity of nanodiamonds per cell (*n* = 30 cells). The black line represents the mean fluorescent intensity bar for each column, (a.u.) stands for arbitrary intensity unit. **** for *p* ≤ 0.0001 and not significant for *p* ≥ 0.05, obtained from non-parametric Kolmogorov–Smirnov tests.

The cellular uptake of raw FNDs was investigated in three types of CNS cells: glioblastoma astrocytes (U87-MG cells; Fig. 4A(i)), neuronal phenotype cells (PC12; Fig. 4A(ii)) and microglia cells (BV-2; Fig. 4A(iii)). Furthermore, cellular uptake of AAL–FNDs (Fig. 4A(iv–vi)), WGA–FND (Fig. 4A(vii–ix)) and TL–FND (Fig. 4A(x–xii)) was investigated in each of the CNS cell types for comparison. Untreated control cells (no diamonds) showed no signal at the emission wavelengths of FNDs (690–800 nm) (Fig. S2†).

Measuring the mean fluorescent intensity of the cells for quantitative comparison (Fig. 4B), raw–FNDs endocytosed by U87-MG and PC12 cell lines were around 8–10 arbitrary unit (a.u.) in contrast to nearly zero by BV-2 cells (Fig. 4A(i–iii) and B). The amount of WGA–FNDs endocytosed by PC12 cells was around 5 times higher than AAL–FND (1 a.u.) and TL–FND (0 a.u.), respectively (Fig. 4A(ii, iv, viii, xi) and B). Microglial cells (BV-2) took in almost no raw–FNDs (0 a.u.) compared to all bioconjugated FNDs and had 50% lower uptake of AAL–FNDs (around 2 a.u.) than TL–FNDs or WGA–FNDs (each around 4 a.u.) (Fig. 4A(iii, vi, ix, xii) and B). No significant increase in raw FNDs endocytosed by U87-MG astrocytes were observed in comparison to AAL–FNDs (both around 8 a.u.), but there was twice and four times more uptake of raw–FNDs (8 a.u.) compared to WGA–FNDs (4 a.u.) and TL–FNDs (2 a.u.) (Fig. 4A(i, iv, viii, x) and B). Additionally, AAL–FND uptake by glioblastoma cells (8 a.u.) was 8 times higher than neurons (1 a.u., *p* ≤ 0.001), and 4 times more than microglial cells (2 a.u., *p* ≤ 0.01) (Fig. 4A(iv–vi) and B). Neuronal phenotype cells (PC12) showed 20% higher uptake of WGA–FNDs (5 a.u.) compared to their uptake of AAL–FNDs (1 a.u.) and TL–FNDs (nearly 0 a.u) (Fig. 4A(vii–ix) and B). Microglial cells took in significantly lower amount of raw–FNDs (almost 0 a.u.) compared to U87-MG (8 a.u., *p* ≤ 0.0001) and PC12 cells (10 a.u., *P* ≤ 0.001) (Fig. 4A(x–xii) and B).

In summary, bioconjugation of FNDs with lectins changed the amount of their uptake by different cell lines. Microglia BV-2 cells had almost no uptake of raw–FNDs while they had the highest uptake of TL–FNDs and WGA–FNDs and very low uptake of AAL–FNDs. The uptake of raw FNDs by BV-2 microglia cells was dramatically increased following LPS treatment, which indicates that inflammation and/or cytokine release is required for raw diamond uptake by these cells (Fig. S6†). PC12 neuronal cells had a higher uptake of raw–FNDs than bioconjugated FNDs, while they showed some uptake of WGA–FNDs and almost no uptake of either AAL–FNDs or TL–FNDs.

### 3.4 Cellular viability and uptake in low temperature conditions

**Fig. 5:**
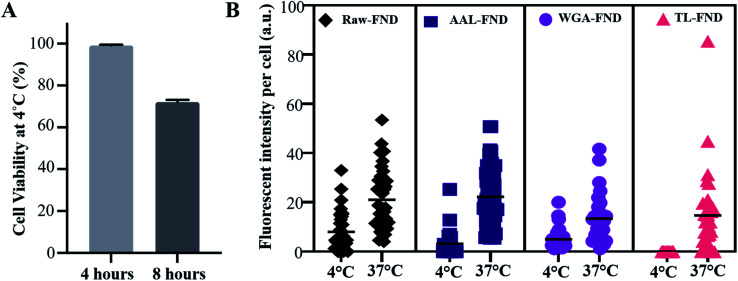
(A) Untreated U87-MG MUSE flow cytometry cell viability test at 4 °C after 4 hours and 8 hours of incubation, showing 70% of U87-MG cells remain viable in this low temperature (*n* = 3 dishes of cultured cells per time point). (B) Comparison of raw and lectin bioconjugated FND (AAL–FND, WGA–FND, TL–FND) uptake by U87-MG astrocyte cells (*n* = 30) following 8 hours of incubation at 4 °C or 37 °C. Imaris software was used to calculate the fluorescent intensity of the mean fluorescent intensity of FNDs of each randomly selected and manually contoured cell. *p* ≤ 0.0001, obtained from non-parametric Kolmogorov–Smirnov tests.

The uptake of raw and bioconjugated FNDs were compared at 4 °C and 37 °C to assess potential differences in active (37 °C) *versus* passive (4 °C) uptake of particles.^63^ In active transport, external molecules are taken in by various cell mechanisms depending on the size and type of the molecules with consumption of energy. At low temperatures such as 4 °C the cell mechanisms are slowed down and the uptake of FNDs is lower than active transport, however this passive transport should not be disrupted. In passive transport no energy exchange is involved, and nanoparticles can pass through the membrane of the cells. Only U87-MG cells were tested because they were the lone cell line in this study with an uptake >0 for raw FND and all three types of lectin bioconjugated FNDs (Fig. 4B). After 4 hours of incubation at 4 °C, U87-MG cells showed 99.5% viability (a rounded average of 3750 viable cells out of 3770 total cells from *n* = 3 replications in 3 plates), which was reduced to an average of 72% viability (a rounded average of 5500 viable cells out of 7500 total cells from *n* = 3 replications in 3 plates) at 8 hours (Fig. 5A). Considering the mean fluorescent intensity bar as a measure for comparison, there was a significantly higher (*p* ≤ 0.0001) uptake of both raw and bioconjugated FNDs at 37 °C compared to 4 °C (Fig. 5B). The uptake of AAL–FNDs at 37 °C (21 a.u) was 7 times higher than at 4 °C (3 a.u) and similarly the uptake of TL–FNDs at 4 °C was negligible while at 37 °C TL–FND uptake (15 a.u) is almost 75% less than that of AAL–FND uptake (∼21 a.u.). WGA–FND uptake at 4 °C (∼5 a.u) was 33% less than its uptake at 37 °C (∼15 a.u.) by astrocytes (Fig. 5B). In all cases the uptake was higher at 37 °C compared to uptake at 4 °C. TL–FND was not taken up at 4 °C by U87-MG cells. Lectin conjugated NDs appeared to be more internalised by active transport rather than passive transport and their transport required more temperature dependant cellular metabolism energy consumption as compared to raw FNDs. The uptake of raw–FNDs at 4 °C (8 a.u) was higher than all bioconjugated FNDs at the same temperature (3 a.u. for AAL–FND, 5 a.u for WGA–FND and 0.003 a.u for TL–FND), while their uptake at 37 °C is almost equal to uptake of AAL–FNDs (∼21 a.u.), having almost 2.5 fold less uptake at lower temperatures.

## 4 Discussion

We have developed a new way of targeting nitrogen vacancy (NV) center nanodiamonds to CNS cells (astrocytes, microglia and neurons) *via* their surface glycan receptors by using lectin conjugation to the nanodiamonds. We utilized FNDs for several reasons including their photostable and bright fluorescence, high uptake and biocompatibility, as well as their physiochemical properties that allow for surface modification and coating with lectins. We observed preferred cell type specificity based on each lectin used and different uptake behaviour for lectin/nanodiamonds compared to raw nanodiamonds. We show here that a variety of lectins (AAL, WGA or TL) that target different glycan receptors (core fucose, sialic acid/GlcNac, poly-*N*-acetyl lactosamine/high mannose *N*-glycan structures on proteins, respectively) can be covalently attached to the surface of nanodiamonds. Each of the CNS cell types investigated here could survive with raw and bioconjugated nanodiamonds at concentrations below 15 μg mL^−1^ with little disruption to cell viability or health^64^ observed at 37 °C. All of the lectins were internalised to slightly different extents by the three cell types but we observed several key cell type dependant preferences. Raw nanodiamonds were highly endocytosed by neurons and astrocytes but microglia cells only endocytosed raw FNDs following LPS stimulated inflammation. In contrast, TL–FNDs were internalized well by microglia cells but were not endocytosed well by neuronal cells. Finally, the uptake of AAL bioconjugated nanodiamonds was significantly higher in human glioblastoma astrocyte cells compared to the other cells types investigated.

We utilized oxidised FNDs in order to apply the common EDC/NHS chemistry method for bioconjugation of lectin proteins to nanodiamonds.^8^ The apparent average size of nanodiamonds in this study increased after bioconjugation with lectins and not at all with PEG. Theoretically, the reason that there is no difference between raw–FNDs and PEG–FNDs could be that raw–FNDs have been aggregated, but the addition of PEG to their surface using EDC/NHS chemistry may have reduced aggregation^65^ while increasing particle size slightly. The molecular weights of AAL, WGA and TL are 72 kilo Daltons (kDa),^66^ 35 kDa (ref. 41) and 71 kDa,^67^ respectively, so the theoretically computed size of the molecules for each lectin should be less than 10 nm.^68^ However, our findings show that bioconjugation of FNDs with lectins adds more than 10 nm to FND–PEG size, possibly due to aggregation. Lectin coatings resulted in a self-aggregating role gathering FNDs into clusters. Since lectins are known for their adhesion ability and many of them agglutinate red blood cells due to their interactions with cell surface carbohydrates, the large surface area of the lectin conjugated FNDs may be due to the formation of aggregated nanodiamond clusters.^69^ Bath sonication was able to temporarily (for about 30 minutes) minimize aggregation of both the raw and bioconjugated FNDs without breaking the particles^70,71^ and our findings demonstrated that sonication did not break the bonds between lectin, PEG and FNDs, in agreement with what has previously been shown following bath sonication at 500 W of WGA conjugated liposomes.^72^ We observed substantial uptake of all FND types (including raw and bioconjugated FNDs) in this study immediately after 10 minutes of bath sonication.

We measured colocalization of lectin–FITC (ex 488 nm, em 516 nm) FNDs containing nitrogen vacancy centers (ex 561 nm, em 690 nm) to confirm that the bioconjugation of lectins to the surface of the diamonds was successful. Nitrogen vacancy FNDs are in a predominantly NV^−^ charge state, as is the case in our material that emit at around the wavelength of 620–690 nm in red (Fig. S7†). It has been shown that FNDs with neutral NV centers emit at around 550–580 nm wavelength, which is in the range for green light emission.^73^ The raw–FNDs without FITC conjugated lectins showed a negligible colocalization of green wavelength emissions in addition to the red emission, supporting that our material does not have a significant proportion of NV centers carrying a neutral charge (10% of the co-localization as seen with FITC lectin bioconjugated FNDs due to possible contaminating emission in the FITC readout from neutrally charged FNDs).

Raw–FNDs and TL–FNDs increased the viability of all cell lines compared to untreated cells as has been reported in previous studies for raw nanodiamonds due to releasing lower amounts of reactive oxygen species.^74,75^ Those experiments were conducted at similar and higher concentrations of up to 100 μg mL^−1^ of raw–FND in neuroblastoma cells and macrophages^74,75^ as well as for *in vivo* studies.^76^ On the other hand, increasing the concentration of WGA–FND or AAL–FND in solution above 15 μg mL^−1^ decreased cell viability. WGA, in contrast to TL, is cytotoxic to a variety of cancer cell subtypes over time, which may be caused by their binding to sialic acid and GlcNAc altering cell cycle functions, or inducing cellular apoptosis and necrosis.^77^ This effect could ultimately be beneficial in utilizing WGA–FND as a nanocarrier for anti-cancer drug delivery. The presence of glycan moieties attached to serum proteins found in cell culture media (albumin, transferrin, aprotinin, fetuin, and fibronectin),^78^ which are targets of AAL and WGA lectins, could also reduce cell viability^79^ as the lectins may interact with the glycoproteins in the media and remove them from nutritional access to the cells. AAL conjugated microparticles have been utilized without cytotoxic effects in gut cells of the small intestine previously although these particles induced cytokine (IL-2, IFN-y, IL-10, IL-5) secretion with increasing concentrations (50–400 μg)^80^ which may have produced a similar inflammation in the brain cells investigated here at the higher (15–30 μg mL^−1^) concentrations that affected cell viability. AAL additionally has structural similarities to *S. typhimurium* LT2 neuraminidase^35^ and is assumed to mimic neuraminidases *in vitro*,^80^ the presence of which can stimulate neuroinflammation in different brain cell subtypes including neurons, microglia and astrocytes depending on which of the four neuraminidase types they are exposed to.^81^

It is well known that AAL binds preferentially to core fucose linked (α-1,6) to *N*-acetylglucosamine^35,36^ and with lower affinity to fucose linked (α-1,2), (α-1,3) and (α-1,4) to *N*-acetyl lactosamine related structures and galactose.^82–85^ Fucosylated glycan structures are upregulated on the surface of many cancer cells^86^ including human glioblastoma multiform (GBM) astrocytes, which has been confirmed by multiple modalities including high performance liquid chromatography (HPLC), lectin blotting, and microscopy with FITC-labelled Ulex europaeus agglutinin-1 (UEA-1) for detection of (α-1,2) fucosylation plus FITC-labelled *Lens culinaris* (LCA) lectin for detection of core (α-1,6) fucosylation.^47,48,87,88^ The upregulation of fucose structures in cancer can be attributed to the fact that fucosylation is essential for many core cell functions such as growth,^89^ proliferation^90^ as well as cell migration,^91^ and these cellular processes occur more in cancer cells than healthy cells.^92–94^ The AAL–FNDs used in the current study were mostly taken up by U87-MG type GBM astrocytes (Fig. 4A(i) and B), which have been reported to have high fucosylation.^95^ However, we observed some AAL–FND uptake by neuronal phenotype cells, which is not surprising as neuronal fucose is reportedly critical in growth,^96^ synaptic interactions, morphology,^97^ as well as learning and memory.^98^ Microglia cells exhibited the least amount of AAL–FND uptake, supporting previous literature demonstrating low core fucosylation in BV-2 cells.^99^

WGA multivalently binds to *N*-acetyl-neuraminic (sialic acid) and *N*-acetyl-d-glucosamine (GlcNAc) containing glycans^38^ while TL can bind to GlcNAc,^39^ as well as to poly *N*-acetyl lactosamine^40^ and high mannose *N*-glycan structures.^41^ We observed some uptake of WGA–FND and TL–FND into every CNS cell type investigated, which is not surprising as GlcNAc structures are abundantly expressed on the surface of most animal cells.^100^ The uptake of WGA–FND was high in every cell line but into neuronal cells in particular, likely because sialic acid type glycans are highly expressed on the surface of neuronal glycosphingolipids and glycoproteins.^101^ WGA is routinely used as a membrane marker for most cell types in fluorescence imaging due to its glycan binding capabilities^102–105^ therefore it is not surprising that WGA was taken in with less specificity into all cell types and this property will be beneficial for cellular endocytosis of nanodiamonds across a variety of cell types for applications such as imaging and drug delivery.^106,107^ TL–FND uptake was lower in neuronal cells, with the most uptake of TL–FNDs seen in microglia cells, in accordance with tomato lectin often being utilized as an *in vivo* marker of microglia cells in the brain for tracking and staining purposes^39,108^ due to binding of oligomannose type *N*-glycans found abundantly on the surface of microglia.^40,109–111^ Our glioblastoma phenotype human astrocytes internalised TL–FNDs, but to a lesser extent than microglia, possibly due to the affinity of TL also for GlcNAc as several lectins such as TL and *Griffonia simplicifolia* lectin II (GSLII) have been used previously to demonstrate binding to GlcNAc on glioblastoma derived cells.^112^

The cellular uptake of nanoparticles is affected by physical properties such as their size, shape, surface area and rigidity.^113^ Nanodiamonds are known for having a high stiffness,^114^ which works in favor of cellular uptake,^115^ but have a large surface area,^1^ which does not favor uptake.^116^ The FNDs used here were heterogeneous in shape and size (20 nm to 260 nm range in size) and the raw–FNDs were endocytosed at high levels by astrocytes and neuronal cells as well as into microglia stimulated by lipopolysaccharide treatment, but not into resting microglia cells. This is in agreement with previous reports that the endocytosis of some nanoparticle types including polystyrene–poly(ethylene glycol) nanoparticles is highly dependent on the disease/inflammation state of microglia^117^ unlike other particles such as quantum dots.

Small nanodiamonds favor higher uptake^118,119^ thus our larger lectin conjugated FND aggregates with a size of 500 nm and above could have more difficulty traveling through the membrane of cells in comparison to the smaller raw–FNDs. Smaller molecules such as dyes can passively enter cells *via* diffusion towards the density gradient without consumption of cellular active energy while larger molecules consume cellular energy and enter cells *via* active transport against membrane resistance.^120^ Active transport is mediated by cell surface-specific receptors such as glycans, the target of lectins, and epidermal growth factor receptor proteins.^121,122^ Low biological temperatures such as 4 °C decrease the speed of cellular metabolism, including endocytosis, and can help to distinguish between active and passive transport.^123,124^ Our findings support that the uptake is shown to increase with temperature based active transport of FNDs into astrocytes, with no passive uptake of TL–FND unlike its active transport level. Increased temperature also differentiated the uptake with the lectin conjugated nanodiamonds showing a greater relative active internalization than at the lower temperature. Active endocytosis is due to clathrin/caveolin-mediated uptake by glycan receptors^121^ and/or is by driven motions (active macropinocytosis) by the cellular cytoskeleton (actin filaments).^113^ These findings suggest that the functional binding capabilities of lectins on the surface of FNDs are retained following our bioconjugation methodologies.

## 5 Conclusions

Our current study presents a novel way of targeted nanodiamond uptake that works by initiating glycan receptor-based endocytosis on the surface of cells, a system that nature readily exploits already for high affinity and efficient viral and bacterial infection in host organisms.^125^ Targeting glycan receptors with nanoparticles bioconjugated to lectins for the purposes of drug delivery or gene delivery is gaining popularity such as utilizing lectins to enhance nasal or oral delivery of medicine,^126–129^ targeting the glycan components of the DNA of HIV patients^130^ and targeting microbes.^131^ Whether glycan targeted therapeutics could face any unforeseen issues in drug delivery is yet to be investigated. Considering the high biocompatibility and superior fluorescence imaging qualities of FND^132–134^ as well as their potential for applications in chemotherapy^135^ and radiotherapy treatments,^136^ our new lectin–FND platform may be able to be adapted easily in future studies for *in vitro*/*in vivo* diagnosis and monitoring of therapeutic effects.

## Funding

This work is funded by the Australian Research Council (ARC) Centre of Excellence Scheme through the Centre of Excellence for Nanoscale BioPhotonics (CE140100003). M. G. is supported by an Australian Government Research Training Program (RTP) Scholarship. L. M. P. is supported by an Australian Research Council Discovery Early Career Research (DECRA) Fellowship (DE180100206) as well as by the Macquarie University Research Centre for Diamond Science and Technology. Z. K. is supported by the Macquarie University Seeding Grant and Restart Grant schemes. P. R. acknowledges support through an Australian Research Council DECRA Fellowship (DE200100279) and a RMIT University Vice-Chancellor's Research Fellowship.

## Author contributions

L. M. P., N. H. P. and M. G. designed the study. M. G., Z. K., P. R., S. D., N. M. C. conducted the experiments in this study. H. A. and T. O. irradiated nanodiamond materials for use in this study. M. G. and Z. K. wrote the manuscript with L. M. P., P. R., N. H. P. and B. G. critically revising the article for important intellectual content.

## Conflicts of interest

The authors declare that the research was conducted in the absence of any commercial or financial relationships that could be construed as a potential conflict of interest.

## Supplementary Material

NA-004-D2NA00036A-s001
