# Supplementary material for: Targeting cell surface glycans with lectin-coated fluorescent nanodiamonds
Source: Nanoscale Adv. 2022 Feb 7;4(6):1551–64. doi: 10.1039/d2na00036a (PMC9418452; doi:10.1039/d2na00036a)
Supplement: NA-004-D2NA00036A-s001 [file NA-004-D2NA00036A-s001.pdf]

Electronic Supplementary Material (ESI) for Nanoscale Advances.
This journal is © The Royal Society of Chemistry 2022

Supplementary information

Targeting cell surface glycans with lectin coated nanodiamonds

Mina Ghanimi Fard[1*], Zahra Khabir[1*], Philipp Reineck[2], Nicole M. Cordina[1], Hiroshi Abe[3], Takeshi Ohshima[3], Sagar Dalal[1], Brant C. Gibson[2], Nicolle H. Packer[1,4], Lindsay M. Parker[1]*

[1] Department of Molecular Sciences and Centre of Excellence for Nanoscale BioPhotonics, Macquarie University, Sydney NSW 2109, Australia

[2] ARC Centre of Excellence for Nanoscale BioPhotonics, School of Science, RMIT University, Melbourne, VIC 3001, Australia

[3] Takasaki Advanced Radiation Research Institute, National Institutes for Quantum and Radiological Science and Technology, Takasaki, Gunma 370-1292, Japan

[4] Institute for Glycomics, Southport, Griffith University, QLD, 4222, Australia

*** Correspondence:**
Dr Lindsay Parker
Lindsay.parker@mq.edu.au

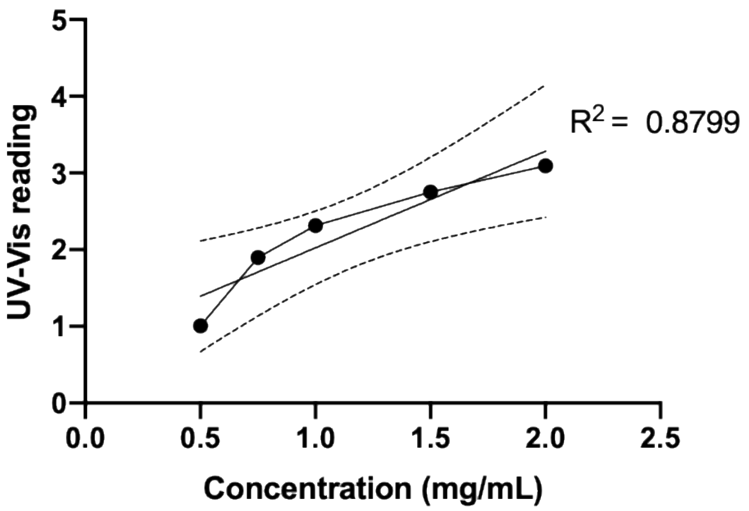

Figure S1. Standard curve from NanoDrop UV-Vis reading data of raw-FNDs at 400 nm wavelength. A NanoDrop ND-2000 Spectrophotometer (Thermo Scientific, Life Technologies) was used to create a standard curve for different concentrations of raw FNDs at 400 nm UV-Vis excitation. The concentrations of 0.5 mg/mL, 0.75 mg/mL, 1 mg/mL, 1.5 mg/mL and 2 mg/mL of raw FNDs were prepared by weighing the FND powder and dilution in DI water. A standard curve of the average reading of each concentration was created using NanoDrop at 400 nm using UV-Vis, and graphed by GraphPad Prism software with a linear regression and an R-squared value of 0.88 (Figure S1). Then, 400nm UV-Vis reading of bioconjugated FNDs were compared to the standard curve to determine the concentration of bioconjugated FNDs. This standard curve with linear regression R^2 ~ 0.88 of known weighed concentration of raw FND water in water was used to determine the concentration of unknown produced bioconjugated FNDs.

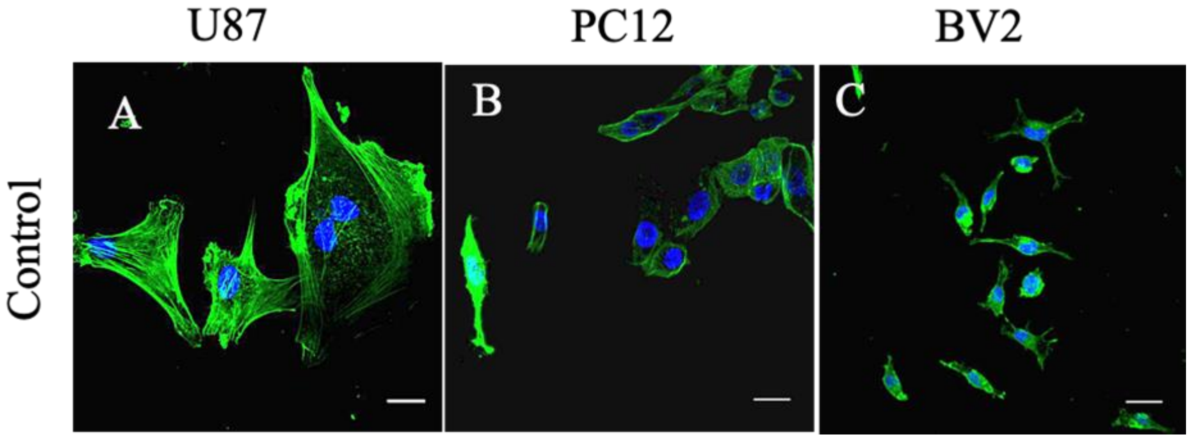

Figure S2. Untreated control brain cells. **(A)** untreated U87-MG cells (astrocyte cells), **(B)** untreated PC12 cells (neuronal phenotype cells), **(C)** untreated BV2 cells (microglia cells) with ActinGreen™ 488 and NucBlue™ stains. Scale bar = 20μm.

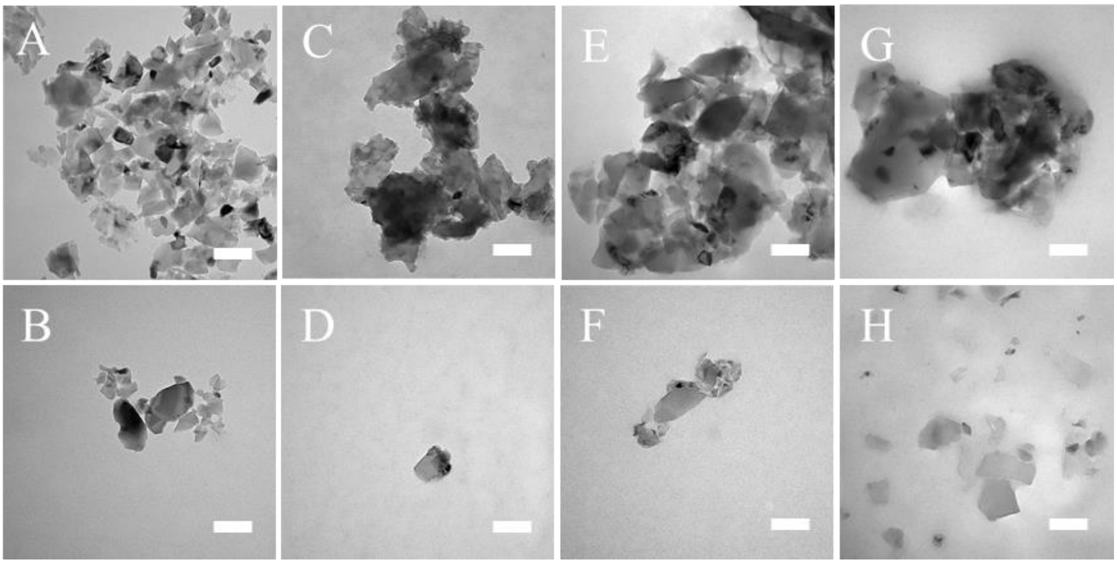

Figure S3. The effect of sonication on the aggregation of FNDs. TEM images of raw FNDs (A, B), AAL-FNDs (C, D), WGA-FNDs (E, F) and TL-FNDs (G, H). The upper row shows aggregation of FNDs before sonication and the bottom row FNDs following 10 min of sonication. Scale bar = 100 nm.

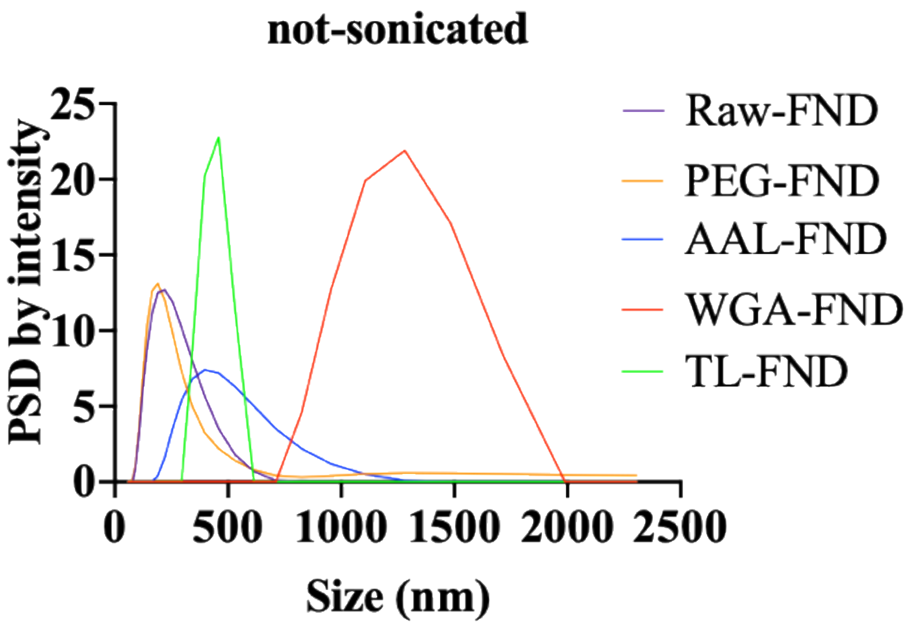

Figure S4. Particle size distribution (PSD) by intensity of colloidal dispersion of highly aggregated raw and lectin bioconjugated FNDs in water without any sonication compared to the particle size of FNDs in Figure 1G.

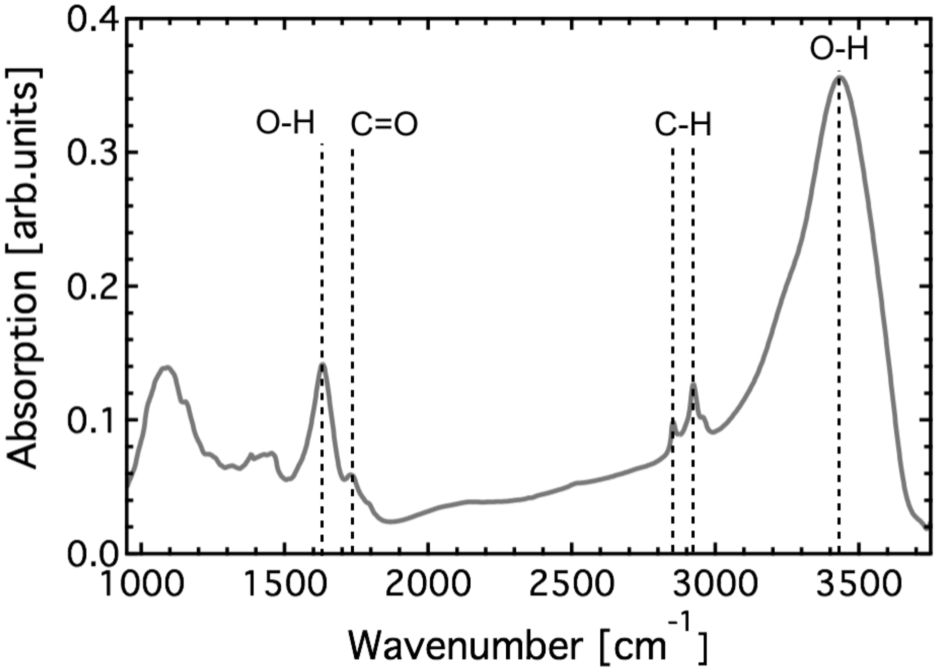

Figure S5. Fourier-transform infrared (FTIR) absorption spectrum of FND particle powder before bioconjugation was recorded with a Frontier spectrometer (Perkin Elmer, USA) fitted with attenuated total reflection (ART) attachment. The spectrum shows several peaks characteristic of O-H, C=O and C-H bonds as indicated by dashed lines.

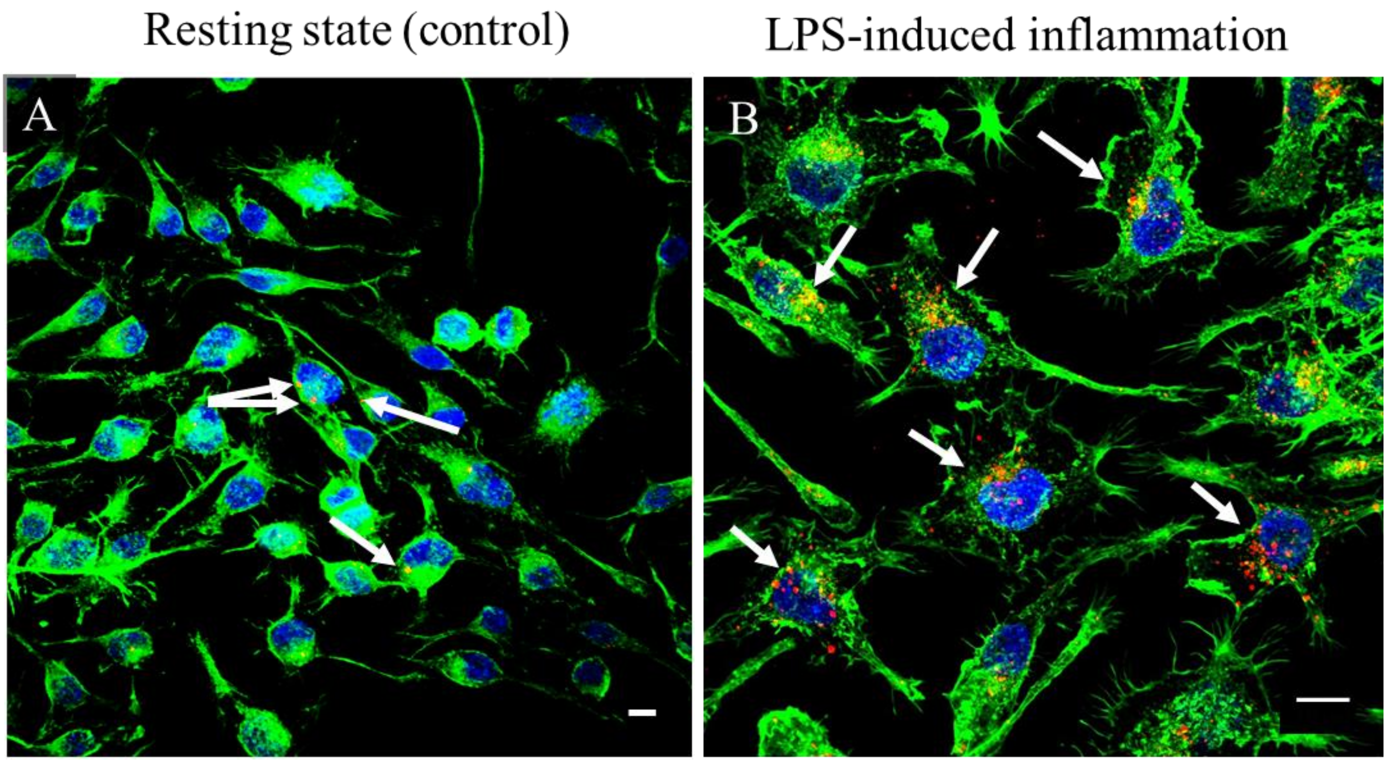

Figure S6. Resting and activated microglia cells 24 hr after application of raw-FNDs. (A) Resting (control) microglia. (B) increased uptake of Raw-FND in LPS treated activated microglia. Scale bar = 10µm.

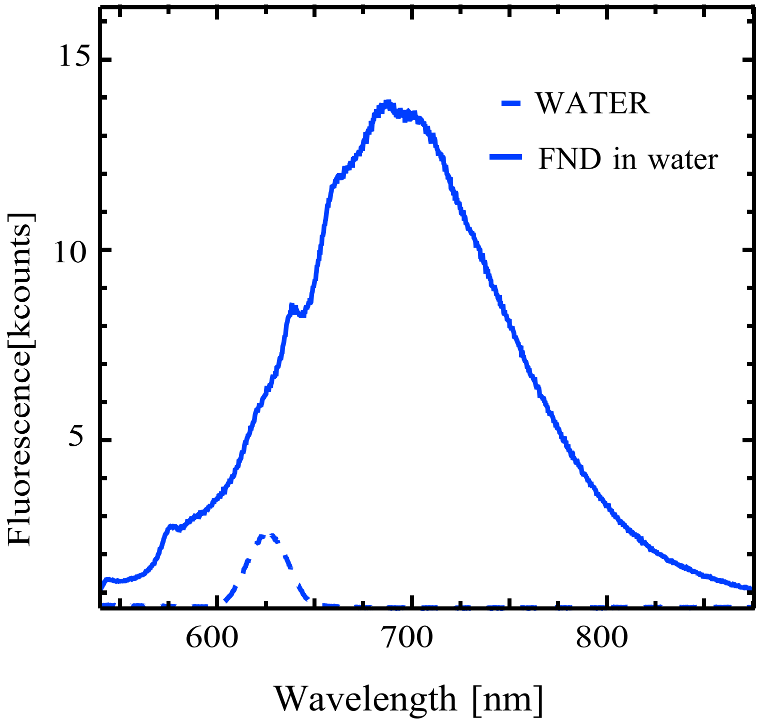

Figure S7. A typical PL spectrum of the 120 nm FNDs suspended in water (0.1 mg/mL). 520 nm laser light was used for excitation. The emission spectrum shows fluorescence from the nitrogen-vacancy center and the characterisitc zero-phonon lines of the neutral (NV^0) and negative (NV^-) charge-state at 575 nm and 637 nm, respectively.
